# Targeted Mutation (R100W) of the Gene Encoding NGF Leads to Deficits in the Peripheral Sensory Nervous System

**DOI:** 10.3389/fnagi.2018.00373

**Published:** 2018-11-13

**Authors:** Wanlin Yang, Kijung Sung, Fengli Zhou, Wei Xu, Robert A. Rissman, Jianqing Ding, Chengbiao Wu

**Affiliations:** ^1^Department of Neurology and Institute of Neurology, Ruijin Hospital, Shanghai Jiao Tong University School of Medicine, Shanghai, China; ^2^Department of Neurosciences, University of California, San Diego, San Diego, CA, United States; ^3^Department of Respiratory Medicine, The Third Affiliated Hospital, Sun Yat-sen University, Guangzhou, China; ^4^Veterans Affairs San Diego Health Care System, San Diego, CA, United States

**Keywords:** hereditary sensory autonomic neuropathy V, nerve growth factor, TrkA, p75 neurotrophic factor receptor, Intraepidermal Nerve Fiber

## Abstract

Nerve growth factor (NGF) exerts multifaceted functions through different stages of life. A missense mutation (R100W) in the beta-NGF gene was found in hereditary sensory autonomic neuropathy V (HSAN V) patients with severe loss of pain perception but without overt cognitive impairment. To better understand the pathogenesis of HSAN V, we generated the first NGF^R100W^ knock in mouse model for HSAN V. We found that the homozygotes exhibited a postnatal lethal phenotype. A majority of homozygous pups died within the first week. Some homozygous pups could ingest more milk and survived up to 2 months by reducing litter size. Whole mount *in situ* hybridization using E10.5 embryos revealed that, compared to wild type, R100W mutation did not alter the gene expression patterns of TrkA and P75^NTR^ in the homozygotes. We also found that the homozygotes displayed normal embryonic development of major organs (heart, lung, liver, kidney, and spleen). Furthermore, the homozygotes exhibited severe loss of PGP9.5-positive intra-epidermal sensory fibers. Taken together, our results suggest that, as with HSAN V patients, the R100W mutation primarily affects the peripheral sensory nervous system in the mouse model. This novel mouse model makes it possible to further study *in vivo* how NGF^R100W^ uncouple trophic function from nociception of NGF.

## Introduction

Nerve growth factor (NGF), a member of the neurotrophin family, exerts multiple actions through different stages of life. During the embryogenesis, NGF, as a potent trophic factor, is necessary for the development and differentiation of sensory and sympathetic neurons (Levi-Montalcini, 1952, 1987). During the adulthood, NGF is important for the survival and functional modulation of basal forebrain cholinergic neurons (BFCNs) and sensory neurons (Hefti, 1986; Mobley et al., 1986; Ritter et al., 1991; Lewin and Mendell, 1993; Pezet and McMahon, 2006; Budni et al., 2015). NGF binds to two distinctive receptors, tyrosine kinase receptor A (TrkA) and the 75 kD neurotrophin receptor (p75^NTR^) (Chao and Hempstead, 1995). Signaling through TrkA mainly exerts trophic function to modulate the neuronal survival and differentiation, while p75^NTR^ may promote cell survival and differentiation by interacting with TrkA or facilitate neuronal apoptosis by interacting with sortilin (Chao and Hempstead, 1995; Bibel and Barde, 2000; Nykjaer et al., 2005; Chen et al., 2009).

Hereditary sensory autonomic neuropathy (HSAN) is a heterogeneous group (type I to V) of neurodegenerative disorders of peripheral nervous system characterized by different degrees of sensory and autonomic dysfunctions (Hilz, 2002). HSAN IV (OMIM # 256800) (Indo, 2001, 2002) and HSAN V (OMIM # 608654) (Einarsdottir et al., 2004; Carvalho et al., 2011) are caused by the mutations in the genes coding for the TrkA and NGF, respectively. HSAN IV patients display mental retardation and severe congenital pain insensitivity, which leads to severe joint destruction and multiple bone fractures (Indo, 2001, 2002). Interestingly, unlike HSAN IV patients, HSAN V patients show similar profound loss of pain sensitivity, but without overt mental retardation (Einarsdottir et al., 2004; Minde et al., 2004). Einarsdottir et al. (2004) first identified the R100W mutation in HSAN V patients located in the Norrbottnian region of Sweden in 2004. The substitution of C to T at nucleotide position 661 (661C > T) in exon 3 of NGF gene located on chromosome 1p11.2-p13.2 result in a substitution of tryptophan (W) for arginine (R) at position 221 in the proNGF polypeptide corresponding to residue R100W in mature NGF (Einarsdottir et al., 2004; Minde et al., 2004). HSAN V patients appear to suffer from loss of pain sensation, but without overt mental retardation, suggesting that the NGF^R100W^ mutation may selectively cause loss of pain function while retaining its trophic function. In a previous study, we already demonstrated that NGF^R100W^ maintained the ability of binding to and activating the TrkA to support neuronal survival and differentiation, but failed to bind and engage p75^NTR^ signaling pathways (Sung et al., 2018).

In this study, we generated a knock in mouse model carrying the naturally occurring NGF mutation, NGF^R100W^, to model HSAN V. This research is the first to directly investigate a mice model for NGF^R100W^ and HSAN V. We found that the beta-NGF^R100W^ knock-in homozygous mice had a pre-mature death within 2 months. Developmental impairments in heart, lung, liver, kidney, and spleen were not observed in homozygous mice. More importantly, there is a severe loss of PGP9.5-positive intra-epidermal sensory fibers in the homozygotes.

## Materials and Methods

### Ethics Statement

All experimental procedures were approved by the Institutional Animal Care and Use Committee of University of California San Diego, Shanghai Jiao Tong University School of Medicine and performed in strict accordance with the NIH Guide for the Care and Use of Laboratory Animals. All tests were carried out using sex- and age-matched mice. In this study, we used heterozygotes and wild type mice that were littermates as control. All mice were housed in individual cages receiving 12 h light/dark cycle at 22°C with access to standard chow and water *ad libitum*.

### Generation of a NGF^R100W^ Knock-in Mouse Model

NGF^R100W^ knock-in mice on a C57BL/6 background were produced by the Model Animal Research Center of Nanjing University (Nanjing, Jiangsu, China). Briefly, a targeting construct containing the A-to-T mutation at the 661 position in exon 3 was generated using classical bacterial artificial chromosome (BAC) recombineering and transfected into ES cells by electroporation. Gene-targeted homologous recombinant clones were identified by Southern blot analysis of genomic DNA isolated from individual neomycin resistant ES cell colonies. Thereafter, appropriately targeted ES cells were microinjected into donor blastocysts followed by implantation into pseudopregnant foster mothers to obtain chimeric mice. Chimeric males were crossed with C57BL/6 females to get heterozygote mice. Heterozygous were interbred to obtain homozygous. Mice were genotyped by PCR. The following PCR primers were used: forward primer 5-GGGGAAGGAGGGAAGACATA-3; reverse primer 5-GATTCCCTTAGGAAGGTTCTGG-3. Band sizes: wild-type band 305 bp; mutant band 435 bp.

### Whole-Mount RNA *in situ* Hybridization to Mouse Embryos

Whole-mount *in situ* hybridization was performed according to previously described procedures (Pizard et al., 2004). Briefly, E10.5 embryos were dissected in cold 1× PBS and immediately fixed with 4% paraformaldehyde (PFA) overnight at 4°C. Embryos were then rinsed in PBT (PBS with 0.1% Triton X-100) to remove fixative, dehydrated by an ascending methanol series and stored in 100% methanol at -20°C. Embryos were rehydrated through a graded methanol series into PBT, digested with 10 μg/ml protease K for 10 min and post-fixed in 4% PFA plus 0.2% glutaraldehyde for 20 min. After prehybridization at 65°C for 2 h’ embryos were incubated overnight in hybridization buffer containing antisense probe at 65°C. Embryos were then washed with a series of washing buffer and blocked in 1× TBST containing 20% sheep serum for 3 h at room temperature. Embryos were incubated overnight with anti-digoxygenin-labeled (DIG)-probes (1:5000; Roche). Following probe incubation, embryos were washed eight times with 1× TBST and rinsed twice for 15 min in fresh NTMT solution. When hybridization signal is optimal, embryos were refixed in 4% PFA.

### Epidermal Innervation

For the detection of epidermal innervation, skin samples were processed as described before (Arcourt et al., 2017). Skins from hind paw footpad of P0 pups were dissected, fixed in methanol/acetone (1:1) for 30 min at -20°C, and then cryoprotected in 30% sucrose overnight at 4°C. Samples were embedded in Tissue-Tek O.C.T compound (Electron Microscopy Sciences, Hatfield, PA, United States) and sectioned into 45 μm. Sections were incubated in 50 mM Glycine for 45 min at room temperature, blocked in 10% goat serum, 1% BSA, and 0.5% Triton X in PBS for 1 h at room temperature, and incubated with rabbit anti-PGP9.5 (Proteintech Group, Inc, Rosemont, IL, United States) with a dilution of 1/300 to 1/500 overnight at 4°C. Sections were then incubated with anti-rabbit Alexa Fluor 594 (Invitrogen, Carlsbad, CA, United States) with a dilution of 1:500 at room temperature for 1 h and counterstained with Hoechst for nuclei for 5 min at room temperature. Images were acquired by confocal microscopy (Leica TCS SP8 Confocal Microscope). For each mouse, 10–15 sections throughout the section thickness (45 μm.) and entire length (200 μm) were analyzed to identify all of the fibers and the intensity of PGP9.5-labeled fibers in the epidermis region was quantified using ImageJ.

### Histopathology

Heart, lung, liver, kidney, and spleen were dissected and fixed in 4% paraformaldehyde for overnight. Samples were then dehydrated in a graded series of alcohol, embedded in paraffin, and sectioned into 4 μm slices. Serial sections were stained with hematoxylin and eosin (HE) and observed under a light microscope (Leica DMi8 Live Imaging Microscope).

### Statistical Analysis

All data were analyzed with GraphPad Prism (GraphPad Software, Inc., La Jolla, CA, United States). The results were presented as mean values ± SEM. Student’s *t*-test was performed for two group comparisons. One-way ANOVA or Two-way ANOVA was performed for multiple comparisons. Differences with *P* < 0.05 was considered statistically significant.

## Results

### Homozygous Mice Are Born Live but Fail to Thrive

**FIGURE 1:**
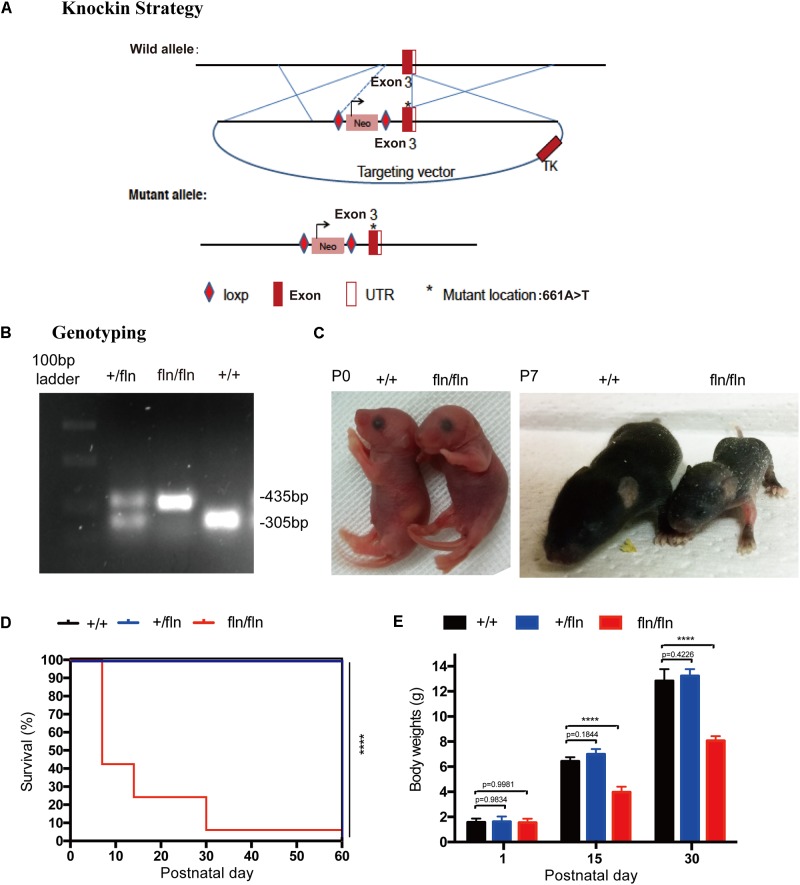
Gene-targeting strategy for generation of NGF^R100W^ knock-in mice model. **(A)** Diagram illustrating the gene-targeting strategy used to produce the NGF^R100W^ knock-in mice in C57BL/6 background. Starred exon 3 represents the mutated exon harboring the A-to-T mutation at the 661 position. **(B)** PCR genotyping of wild-type (+/+), the heterozygote (+/fln) and the homozygote (fln/fln): wild-type band: 305 bp, mutant band: 435 bp. **(C)** fln/fln mice appeared normal at birth (P0), but failed to grow during early postnatal life (e.g., at P7) compared with +/+ littermates. **(D)** Survival curves for +/+ (*n* = 20), +/fln (*n* = 20), and fln/fln (*n* = 20) mice. fln/fln mice died within 2 months. **(E)** The body weight growth curves for +/+ (*n* = 6), +/fln (*n* = 6), and fln/fln (*n* = 6) mice. fln/fln mice gained weight more slowly than their littermate. ^∗∗∗∗^*p* < 0.0001, one way ANOVA test followed by Tukey’s multiple comparisons test.

To shed light on the role of NGF^R100W^ mutation in the pathogenesis of HSAN V, we generate NGF^R100W^ knock-in mice on C57BL/6 background through the knock-in strategy (Figure 1A). Briefly, a targeting construct containing the A-to-T mutation at the 661 position, which leading to the amino acid substitution R to W at 100 position of mature NGF, was electroporated into ES cells. The positive ES clones containing the mutation were selected and microinjected into donor blastocysts followed by implantation into pseudopregnant foster mothers to obtain male chimeric mice. Chimeric males were mated with C57BL/6 females to obtain heterozygotes (+/fln). +/fln mice were interbred to obtain homozygous (fln/fln). The +/fln and fln/fln mice were genotyped by PCR using tail DNA (Figure 1B).

The fln/fln mice exhibited a postnatal lethal phenotype (Figure 1D). A huge majority of fln/fln pups died within the first week of life. fln/fln mice exhibited smaller milk spot in the stomach by visual inspection, indicating they could not ingest normal quantities of milk. By moving littermates that has bigger milk spot to a wild-type (+/+) surrogate mother to reduce litter size, some fln/fln mice could ingest more milk and survived up to 2 months. The fln/fln mice were born with normal body weight and size, but grew more slowly than their +/+ and +/fln littermates. As shown in Figure 1C, newborn fln/fln pup had comparable body size to the +/+ littermates, but became significantly smaller on P7. By 15 and 30 days of age, the body weight of fln/fln pups was remarkably lower, only 61.6 and 62.8% of that of +/+ littermates, respectively (Figure 1E). Whereas, the +/fln mice exhibited normal body weight and were indistinguishable from the wild-type (+/+) littermates by observing gross morphology or spontaneous behavior (Figure 1E). These results indicated that fln/fln mice were born live with normal weight, but failed to thrive.

### NGF^R100W^ Mutation Do Not Affect the Gene Expression Patterns of TrkA and P75^NTR^

**FIGURE 2:**
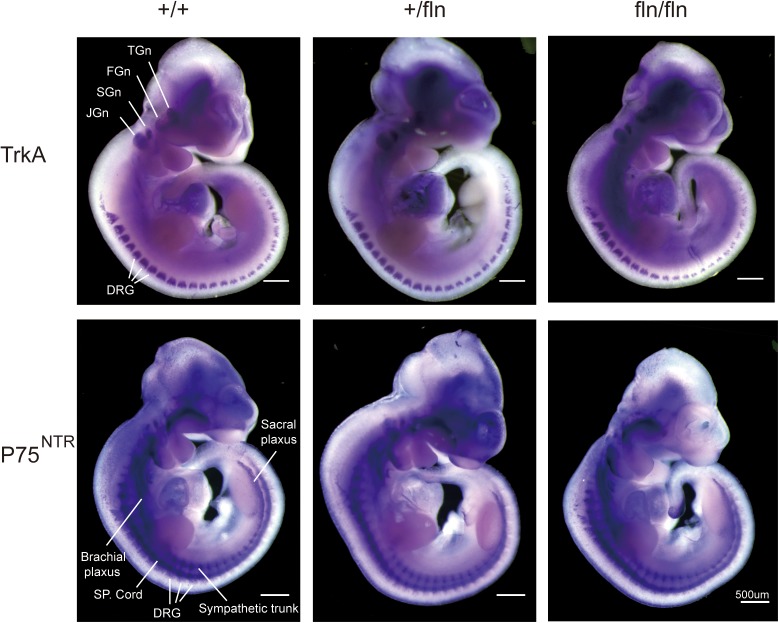
Expression of TrkA and P75^NTR^ is normal in homozygous. Whole-mount *in situ* hybridization using antisense riboprobe for TrkA and P75^NTR^ mRNA was performed on +/+ (*n* = 3), +/fln (*n* = 3), and fln/fln (*n* = 3) embryos at E10.5. TGn, trigeminal ganglion; FGn, facial ganglion; SGn, superior ganglion; JGn, jugular ganglion; DRG, dorsal root ganglion. Bar = 500 μm.

The NGF signaling is mediated by two known receptors (Chao and Hempstead, 1995), TrkA and p75^NTR^, we next assessed whether the gene expression patterns of TrkA and P75^NTR^ in +/fln and fln/fln E10.5 embryos exhibited defects using whole mount *in situ* hybridization. As shown in Figure 2, TrkA was highly expressed in cranial and spinal sensory ganglia, including dorsal root ganglions, trigeminal, facial ganglion, and superior ganglion. Whereas, P75^NTR^ was expressed in wider range including the peripheral nerve trunks, dorsal root ganglia, and spinal cords. There was no obvious difference in gene expression patterns of TrkA and P75^NTR^ among +/+, +/fln, and fln/fln embryos mice do not display gross developmental defects.

### Homozygous Embryos Do Not Display Gross Developmental Defects

**FIGURE 3:**
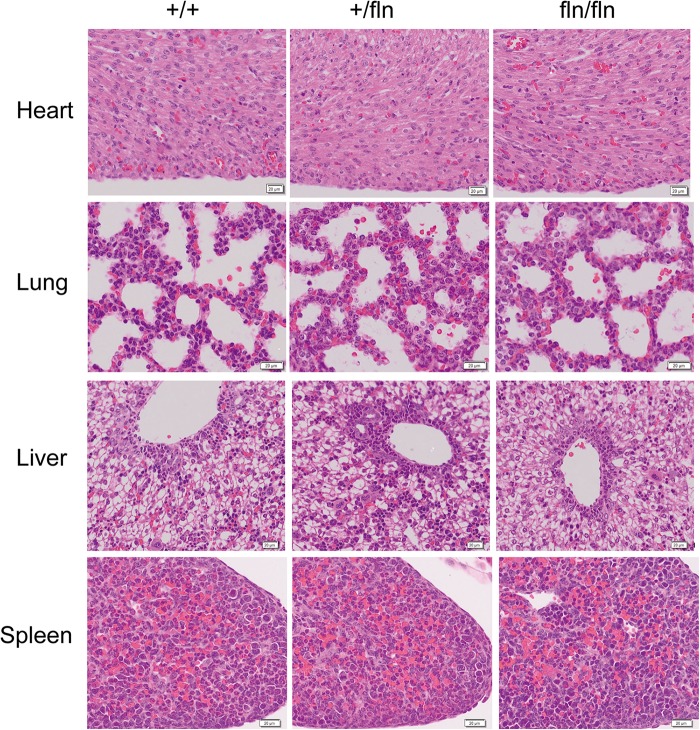
No obvious histological abnormalities were observed in heart, lung, liver, and spleen of homozygous. Representative sections of H&E staining on heart, lung, liver, and spleen samples from +/+ (*n* = 3), +/fln (*n* = 3), and fln/fln (*n* = 3) embryos at E18.5. Bar = 20 μm.

Although P0 fln/fln pups were born live and indistinguishable from their +/+ littermates in size and weight, a huge majority of fln/fln pups died within the first week of life. As the fln/fln mice exhibited a postnatal lethal phenotype, we then analyzed the tissue morphology more closely to assess whether fln/fln exhibited embryonic developmental defects. H&E staining of heart, lung, liver, and spleen tissues from +/+, +/fln, and fln/fln embryos at E18.5 was performed (Figure 3). Histological comparison failed to reveal any striking abnormalities in major organs of +/fln and fln/fln embryos.

**FIGURE 4:**
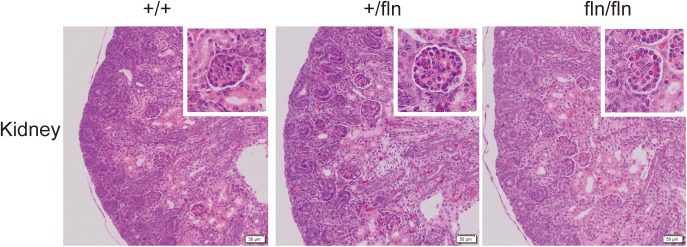
No kidney defects were detected in homozygous. Representative sections of H&E staining on kidney samples from +/+ (*n* = 3), +/fln (*n* = 3), and fln/fln (*n* = 3) embryos at E18.5. Bar = 50 μm at low magnification, Bar = 10 μm at higher magnification.

Previous research on NGF^R100W^ revealed that R100W mutation selectively disrupted binding of NGF to p75^NTR^, while it retained the ability of binding to TrkA (Covaceuszach et al., 2010; Sung et al., 2018). The p75^NTR^ is transiently synthesized in embryonic kidney (Sariola et al., 1991). A study reported that anti-sense oligonucleotide inhibition of p75^NTR^ expression in organ culture inhibited kidney morphogenesis, indicating that p75^NTR^ signaling played an important role in embryonic development of the kidney (Sariola et al., 1991). We next detected whether fln/fln embryos displayed developmental defects of the kidney. As illustrated in Figure 4, fln/fln embryos did not show obviously difference from their +/+ and +/fln littermates.

**FIGURE 5:**
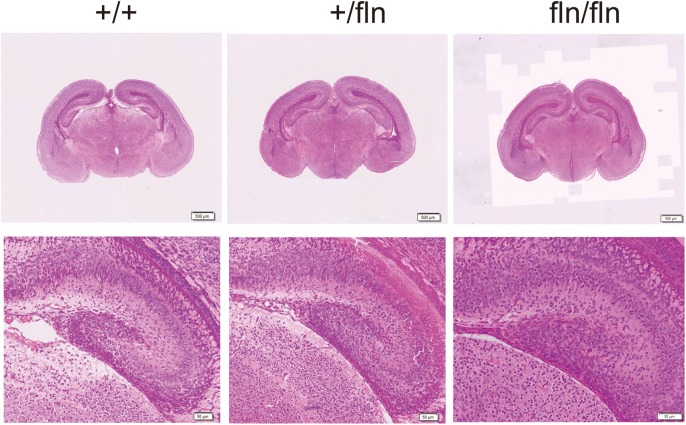
No obvious histological abnormality was detected in homozygous. Representative sections of H&E staining on hippocampus samples from +/+ (*n* = 3), +/fln (*n* = 3), and fln/fln (*n* = 3) embryos at E18.5. Bar = 500 μm at low magnification, Bar = 50 μm at higher magnification.

Nerve growth factor is produced in the hippocampus throughout life and essential for hippocampal plasticity and learning (Conner et al., 2009). We next examined whether fln/fln embryos displayed developmental defects of hippocampus. As illustrated in Figure 5, no obvious differences in the pyramidal cell layer in the hippocampus are discernable between +/+, +/fln, and fln/fln littermates at E18.5 of age. These findings indicated that homozygous embryos display normal embryonic development of major organs were indistinguishable from those of wild-type littermates in size, weight, and general appearance.

### Homozygous Mice Exhibited Severe Loss of PGP9.5-Positive Intra-Epidermal Sensory Fibers

**FIGURE 6:**
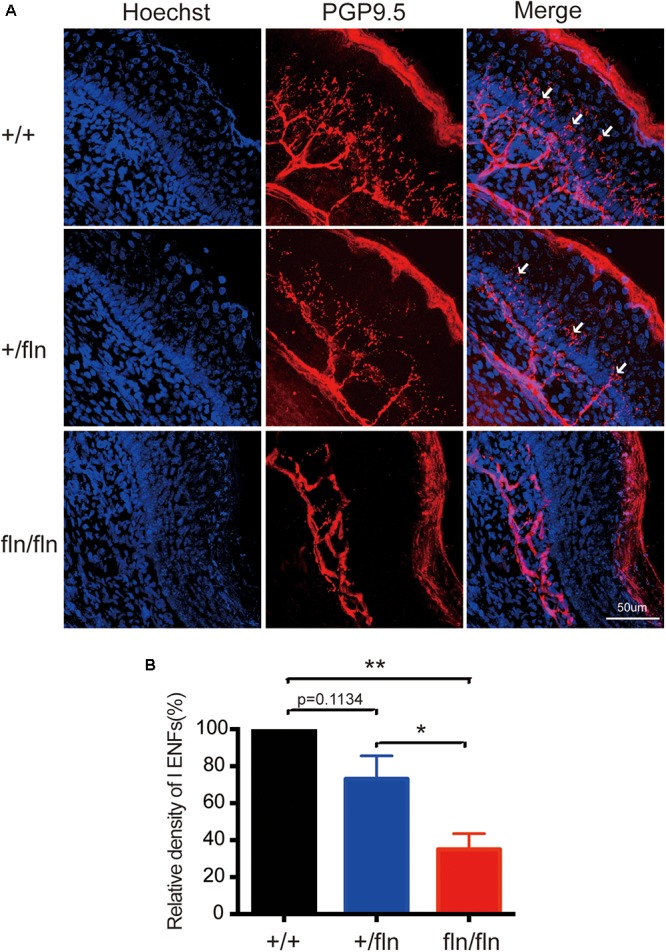
Loss of PGP9.5-positive intra-epidermal sensory fibers (IENF) in the footpad skin in homozygous. **(A)** Confocal microscopic images from sections through the hindpaw footpad skin from +/+ (*n* = 3), +/fln (*n* = 3), and fln/fln (*n* = 3) P1 mice stained with pan-nerve fiber marker PGP9.5 (red) and nuclear marker Hoechst (blue) to visualize skin cells. The arrowhead indicates the free nerve endings, **(B)** Quantification of PGP9.5-positive IENF intensities. Mean ± SEM, ^∗^*p* < 0.05, ^∗∗^*p* < 0.01, one way ANOVA test followed by Tukey’s multiple comparisons test.

Nerve growth factor is important for the development and differentiation of sensory neurons during embryogenesis (Levi-Montalcini, 1987; Li et al., 2017; Sun et al., 2018). Painful signals on the skin were transmited to the spinal cord and brain by myelinated Aδ and unmyelinated C sensory nerve fibers (Adrienne and Dubin, 2010). Aδ and C nerve fibers are significantly decreased in HSAN V patients (Einarsdottir et al., 2004; Minde et al., 2004, 2006). We then investigated whether +/fln and fln/fln pus displayed severe sensory nerve fibers loss in hind paw skin. The PGP9.5 antibody was used to examine intra-epidermal sensory fibers (IENFs), including Aδ and C nerve fiber. The staining of hind paw skins revealed that the IENFs appeared significantly reduced in fln/fln pups compared with +/+ and +/fln pups (Figure 6). There was no significant differrence between +/+ and +/fln pups.

## Discussion

HSAN is a heterogeneous group (type I to V) of rare, clinically and genetically peripheral neuropathies characterized by different degrees of sensory and autonomic dysfunctions (Capsoni, 2014). Genetic mutations responsible for HSANs have been demonstrated. In the cases of HSAN IV (OMIM # 256800) (Indo et al., 2001; Indo, 2002) and HSAN V (OMIM # 608654) (Einarsdottir et al., 2004; Minde et al., 2004), which are caused by mutations in the genes coding for the TrkA and NGF respectively, both of them are all involved in NGF signaling pathway abnormality. Interestingly, HSAN IV patients display severe congenital pain insensitivity and mental retardation, while HSAN V patients show similar pain insensitivity, but without overt mental retardation (Capsoni, 2014). The mutation 661C > T in NGF gene of HSAN V patients located in the Norrbottnian region of Sweden was first identified by Einarsdottir et al in 2004 (Einarsdottir et al., 2004). This mutation causes a substitution of tryptophan (W) for arginine (R) at position 221 in the proNGF polypeptide corresponding to residue R100 in mature NGF. The different clinical manifestations between HSAN IV and HSAN V patients motivated us to generate NGF^R100W^ knock-in mice to better define the pathogenesis of HSAN V. This research is the first to directly investigate a mice model for NGF^R100W^. We found that the homozygous mice exhibited a postnatal lethal phenotype. Most homozygous pups died within the first week of life. Some homozygous pups could ingest more milk and survived up to 2 months by reducing litter size. The homozygous and wild type embryos showed similar gene expression patterns of TrkA and P75^NTR^. The homozygous mice displayed normal embryonic development of major organs. Furthermore, the homozygous mice exhibited severe loss of PGP9.5-positive intra-epidermal sensory fibers.

In 2004, Einarsdottir et al first described the R100W mutation in HSAN V patients located in the Norrbottnian region of Sweden Minde et al. (2004). It has been shown previously that HSAN V homozygous patients showed a small reduction of myelinated Aδ nerve fibers and a severe reduction of unmyelinated C nerve fibers, while this reduction was milder in heterozygous carriers (Einarsdottir et al., 2004; Larsson et al., 2009). Consistent with HSAN V patients, we confirmed that homozygous mice exhibited severe loss of intra-epidermal sensory fibers in hind paw skin, including Aδ and C nerve fibers. In many ways, our NGF^R100W^ homozygotes resemble the NGF knockout homozygotes mice in their severe sensory phenotypic presentation. Crowley et al. generated the first transgenic NGF knockout mice model in 1994 and found that homozygotes appeared to reveal somewhat loss of sensory fibers in the skin (Crowley et al., 1994). The similarity between NGF^R100W^ knock-in and NGF knockout homozygotes indicated the deficits in the NGF^R100W^ protein synthesis and secretion or function and structure. Indeed, Larsson et al already demonstrated that proNGF^R100W^ was predominantly accumulated in the cell lysate and the secretion of mature NGF^R100W^ was markedly reduced (Larsson et al., 2009). The accumulation of proNGF^R100W^ did not appear to be due to an inability of the convertases, such as furin and plasmin, to cleave the pro-protein to mature protein, but was related to the intracellular localization that would prevent processing and secretion of mature NGF^R100W^ (Larsson et al., 2009). The reduced secretion of mature NGF^R100W^ may lead to deficiency of mature NGF in innervated targets, which could explain the severe loss of sensory fibers in HSAN V patients and NGF^R100W^ knock-in mice. One the other hand, structural insights into the R100W mutation in NGF protein also found that residue R100 is not directly participated in the interface of TrkA, but it involved in the p75^NTR^ interaction surface, suggesting that R100 mutation selectively disrupted the binding of NGF to p75^NTR^ (Covaceuszach et al., 2010). Further studies demonstrated that R100W mutation did not affect NGF binding to TrkA, while it abolished NGF binding to p75^NTR^ (Capsoni et al., 2011). Our previous study also demonstrated that NGF^R100W^ maintained the ability of binding to and activating the TrkA to support neuronal survival and differentiation (Sung et al., 2018), which could explain why the cognitive impairment of HSAN V patients appeared very limited or absent.

Hereditary sensory autonomic neuropathy V homozygous patients can survive to adulthood, but they do acquire multiple fractures and being burned during childhood due to the lack of deep pain sensations and increased cold and heat thresholds (Einarsdottir et al., 2004; Minde et al., 2004; Larsson et al., 2009). However, none of those patients displayed systemic diseases related to heart and lungs (Einarsdottir et al., 2004). Whereas, in the cases of our NGF^R100W^ knock-in mice, a huge majority of homozygous mice died within the first week of life, which led us to examine whether the homozygous mice exhibited embryonic developmental defects. Histological comparison of heart, lung, liver, kidney, and spleen tissues failed to reveal any striking abnormalities in major organs of homozygous embryos, indicating that postnatal lethality of homozygous mice was not caused by embryonic developmental defects. Observations during the early postnatal days showed that homozygous mice displayed smaller milk spot in the stomach, indicating they could not ingest normal quantities of milk. Some homozygous mice could ingest more milk and survived up to 2 months by reducing litter size. Therefore, we speculated that postnatal lethality of homozygous mice might be related in part to ineffective ingestion. Furthermore, as homozygous mice displayed severe loss of IENFs in skin, the deficits in the sensory nervous system might be one of the leading causes of death of homozygous mice. Before the birth, embryos are isolated in the uterus and obtain nutrients from their mothers. After birth, the environment is drastically change and the newborn pups start to receive external sensory stimuli and need to interact with other littermates and mother to keep warm and obtain milk (Weber et al., 2013; Toda and Kawasaki, 2014). The sensory nervous system deficits may affect the mother-pup and pup–pup interactions (Toda and Kawasaki, 2014). During the first week of life, we also found that homozygous mice often stayed outside the nest. After birth, the newborn pups are totally dependent on their mother for thermoregulation and nutrition, and could die from hypothermia or starvation (Terrien et al., 2011). The maternal behavior in mouse and human is different. Mouse mother sometimes tend to cull if a pup is too weak and likely to die, while human are the opposite and will try their best to keep the babies healthy, which may explain why the HSAN V homozygous patients can survive to adulthood and our homozygous mice exhibit postnatal lethality. Furthermore, as lack of more specific or detailed histological analysis, we cannot rule out the possibility that other pathological changes outside the nervous system contribute to the postnatal lethality of homozygous. Further studies need to be performed to investigate the pathological changes in other tissues.

Taken together, we generated the first NGF^R100W^ knock in mouse model to study the pathogenesis of HSAN V. We found that NGF^R100W^ knock-in homozygotes had a pre-mature death within 2 months. However, The developmental impairments in heart, lung, liver, kidney, and spleen were not observed in homozygous mice. More importantly, we demonstrated that, as with HSAN V patients, the homozygous mice displayed severe loss of sensory fibers in the skin. This novel mouse model makes it possible to further investigate the pathogenesis for HSAN V. More importantly, it provides a better tool to further study *in vivo* how NGF^R100W^ uncouple trophic function from nociception of NGF.

## Author Contributions

WY, KS, JD, and CW designed and performed the study and wrote the manuscript. RAR helped with the design and histological analysis. WX and FZ performed analysis of PGP9.5/IENFs staining experiments.

## Conflict of Interest Statement

The authors declare that the research was conducted in the absence of any commercial or financial relationships that could be construed as a potential conflict of interest.
